# Effect of quorum quenching enzyme YtnP on immune response and intestinal microbiota in *Aeromonas hydrophila*-infected grass carp (*Ctenopharyngodon idella*)

**DOI:** 10.3389/fmicb.2026.1853827

**Published:** 2026-08-24

**Authors:** Huifang Luo, Zhen Zhao, Kaiyi Zhou, Tianyi Lv, Yuan Wang, Anli Hu, Zengfu Song

**Affiliations:** 1 National Demonstration Center for Experimental Fisheries Science Education, Shanghai Ocean University, Shanghai, China; 2 National Pathogen Collection for Aquatic Animals, Shanghai Ocean University, Shanghai, China; 3 College of Fisheries and Life Science, Shanghai Ocean University, Shanghai, China; 4 China-ASEAN "The Belt and Road" Joint Laboratory of Marine Culture Technology (Shanghai), Shanghai, China

**Keywords:** *Aeromonas hydrophila*, *Ctenopharyngodon idella*, quorum quenching enzyme, quorum sensing, YtnP

## Abstract

*Aeromonas hydrophila* induces tissue swelling, necrosis, ulcers, and hemorrhagic septicemia in grass carp, causing significant economic losses. Quorum sensing, a communication mechanism among microorganisms, plays a key role in microbial ecological competition and dynamic equilibrium. Quorum quenching has emerged as a hot spot in anti-bacterial infection research. In this study, grass carp (*n* = 75 per group) were intraperitoneally injected with *A. hydrophila* and the quorum quenching enzyme YtnP. The expression levels of immune-related genes were assessed by qPCR. The intestinal microbiota was sequenced using the Illumina HiSeq 6,000 platform, with subsequent bioinformatics analysis. Co-treatment with YtnP significantly improved the survival rate of grass carp infected with *A. hydrophila*, increasing the survival rate from 57% in the AH group (*A. hydrophila*-infected only) to 87% in the AY group (co-administration of *A. hydrophila* and YtnP). Analysis of immune-related gene expression showed that the AH group exhibited early upregulation of the pro-inflammatory IL-1β and TNF-*α*, whereas the AY group showed increased expression of IFN-γ1, IL-6, IL-15, and IL-10, suggesting that YtnP contributed to immune regulation. Microbiota analysis revealed *A. hydrophila* infection caused dysbiosis, with a significant increase in the relative abundance of *Proteobacteria* and significant decreases in *Fusobacteria* and *Actinobacteria*. YtnP treatment significantly restored the abundance of *Fusobacteria* and *Actinobacteria* and altered predicted metabolic pathways related to aromatic compound metabolism and enterobactin biosynthesis, suggesting a potential role in modulating intestinal microbial community structure during *A. hydrophila* infection. By blocking bacterial virulence signal transmission and restructuring the intestinal microbiota toward probiotic enrichment, YtnP enhanced the host’s innate immune response to *A. hydrophila* and restored host-microbiota homeostasis. This study establishes YtnP as a potential antimicrobial agent for sustainable aquaculture and provides a theoretical foundation for the quorum quenching-based immune-microbiota interaction mechanism in fish.

## 1 Introduction

*Aeromonas hydrophila*, as an opportunistic pathogen, exhibits pathogenicity closely associated with diverse virulence factors, including hemolysins, proteases, and biofilm formation-associated genes, which can induce tissue lesions and high mortality rates in aquatic farming animals (Abdella et al., 2024; Abreu et al., 2018). *A. hydrophila* infection causes intestinal inflammation in grass carp, along with hepatic lesions, splenomegaly, and nephromegaly, ultimately progressing to septic shock and lethal consequences (Guo et al., 2023; Salifu et al., 2023; Wang et al., 2017). Such infections result in substantial economic losses to aquaculture industries and may pose public health risks through zoonotic transmission pathways (Song et al., 2014).

The dynamic interaction network formed through long-term co-evolution between fish intestinal microbiota and the host immune system plays a pivotal role in maintaining host homeostasis. Gut commensal microbiota preserves organismal health by competitively occupying ecological niches, secreting antimicrobial peptides to suppress pathogen proliferation, and modulating host metabolism and immune responses (Diwan et al., 2022; Dong et al., 2017; Zhang et al., 2024). The intestine dynamically regulates host immune functions by distinguishing commensal microbiota from pathogens. Host immune cells express pattern recognition receptors (PRRs), such as Toll-like receptors (TLRs) and nucleotide-binding oligomerization domain-like receptors (NLRs), which specifically recognize microbe-associated molecular patterns (MAMPs) (Hill and Diehl, 2018). Upon recognition of commensal bacteria, PRRs activate signaling transduction cascades including the Nuclear Factor-κB (NF-κB) pathway and Mitogen-activated Protein Kinase (MAPK) pathway, thereby inducing moderate expression of pro-inflammatory cytokines to initiate innate immune responses. Furthermore, gut microbiota maintains immune homeostasis by enhancing intestinal barrier integrity and regulating immune cell activity. Notably, dysbiosis-induced reduction of commensal bacteria decreases short-chain fatty acids (SCFAs) levels, impairing immune tolerance, while pathogen overgrowth triggers excessive inflammation through persistent activation of PRRs signaling pathways, leading to host immune dysregulation (Fénero et al., 2021). Current strategies to restore and maintain microbial balance include probiotic and prebiotic supplementation to competitively inhibit pathogens and promote beneficial colonization or phage cocktail therapy to specifically lyse pathogens without affecting commensals (Federici et al., 2022; Soltani et al., 2019). Additionally, the application of probiotics with quorum-quenching capabilities, which express quorum-quenching enzymes to disrupt microbial communication by suppressing signal molecule production, replication, and detection, effectively reduce virulence factors in multiple significant fish pathogens (Amrullah et al., 2024; Ghanei-Motlagh et al., 2021a, b; Lubis et al., 2024).

Quorum sensing (QS) is a ubiquitous intercellular communication mechanism in bacteria, centered on the secretion and perception of autoinducers, such as diffusible signal factor (DSF) and acyl-homoserine lactones (AHLs) in Gram-negative bacteria, autoinducing peptides (AIPs) and interspecies signaling molecule AI-2 in Gram-positive bacteria, to monitor population density and synchronize gene expression (Zhu et al., 2023). When bacterial density reaches a critical threshold, accumulated signal molecule concentrations trigger population-wide phenotypic changes, such as virulence factor secretion and biofilm formation. For instance, the Agr system in *Staphylococcus aureus* regulated *α*-hemolysin expression to promote abscess formation in mice (Rauch et al., 2012). At the same time, *Pseudomonas aeruginosa* employed the LasI/LasR and RhlI/RhlR systems to coordinate biofilm formation and pyocyanin production (Davies et al., 1998).

Quorum quenching enzymes disrupt bacterial communication by specifically degrading QS signal molecules, thereby inhibiting virulence gene expression and biofilm formation (Fetzner, 2015). Unlike traditional antibiotics, these enzymes target bacterial signaling systems rather than directly killing bacteria, offering a novel strategy for anti-virulence therapy. Our research group previously cloned and characterized the *N*-acyl homoserine lactonase YtnP from *Bacillus licheniformis* T-1, demonstrating its ability to significantly suppress *A. hydrophila* virulence gene expression and biofilm formation while enhancing *Carassius auratus* resistance to *A. hydrophila* infection (Peng et al., 2021). However, the impact of quorum quenching mechanisms on host immune regulation and the dynamic intestinal microbiota ecosystem remains unclear. This study investigates the effects of exogenous YtnP supplementation on immune function and gut microbiota composition in grass carp, aiming to elucidate the relationship between quorum quenching activity and host-microbiota homeostasis, thereby providing a theoretical foundation for its scientific application in sustainable aquaculture.

## 2 Materials and methods

### 2.1 Animal and bacterial material, culture conditions

*Aeromonas hydrophila* ATCC7966 was obtained from the American Type Culture Collection (ATCC) and grown in LB medium at 30 °C for 20 h. The median lethal dose (LD_50_) was determined as 7 × 10^6^ CFU/mL at a standardized injection volume of 100 μL. Subsequently, *in vivo* validation established 3 × 10^6^ CFU/mL as the optimal challenge dose in grass carp models.

The recombinant expression strain *Escherichia coli* BL21(DE3)-T-easy-ytnP carrying the pEASY-Blunt E1-ytnP plasmid, which encodes the N-acyl homoserine lactonase YtnP (Genbank: MK360859.1) from *Bacillus licheniformis* T-1 fused with a C-terminal 6 × His tag, was constructed. The recombinant YtnP protein was expressed in *E. coli*, refolded, and purified. Prior to administration, the purified protein was diluted to 25 μg/mL in size-exclusion chromatography buffer (20 mM Tris–HCl, pH 8.0, 50 mM NaCl) for experimental injections.

Juvenile grass carp (28 ± 1 g/fish, *n* = 300) sourced from the University’s Coastal Science Education Base were maintained in the National Aquatic Animal Pathogen Repository recirculating aquaculture system under controlled conditions: water temperature 25 ± 2 °C, pH 7.5 ± 0.5, with twice-daily feeding (1% body weight per feeding at 08:00 and 16:00). All animal procedures were conducted following protocols approved by the Animal Ethics Committee of Shanghai Ocean University.

Fish were randomly allocated into four experimental groups, with 75 fish per group (25 fish per replicate, three biological replicates). Each fish was intraperitoneally injected with 100 μL of the corresponding treatment. The Control group received size-exclusion chromatography buffer (20 mM Tris–HCl, pH 8.0, 50 mM NaCl); the AH group received an *A. hydrophila* suspension (3 × 10^6^ CFU/mL in buffer); the YtnP group received recombinant YtnP protein (25 μg/mL in buffer); and the AY group received a mixture of *A. hydrophila* and recombinant YtnP protein. Fish were anesthetized with 40 mg/L MS-222 (tricaine methanesulfonate) prior to injection. All injections were conducted aseptically using sterile 1 mL syringes via the intraperitoneal route.

### 2.2 Sample collection

Mortality rates were recorded daily post-injection. Prior to sampling, fish were fasted for 24 h to minimize the interference of food residues. The entire intestine was dissected, and the foregut, midgut, and hindgut segments with their luminal contents were aseptically collected at 1, 3, 5, and 7 days post-injection. At each time point, six fish per group (two from each of the three replicates) were sampled for high-throughput 16S rRNA amplicon sequencing analysis. Tissue samples were homogenized, snap-frozen in liquid nitrogen, and preserved at −80 °C for sequencing.

Concurrently, four fish per group (distributed across the three replicates, at least six biological samples per group) at each time point were sampled for RNA extraction and qPCR analysis. Intestine tissue, spleen, and head kidney samples collected from the same fish were mechanically homogenized in RNA stabilization buffer for subsequent Quantitative Polymerase Chain Reaction analysis (qPCR).

### 2.3 qPCR analysis of immune-related gene expression

The expressions of non-specific immune genes interleukin-15 (IL-15), interleukin-β1 (IL-1β), interleukin-6 (IL-6), interleukin-8 (IL-8), interleukin-10 (IL-10), transforming growth factor-β1 (TGF-β1), interferon-γ1 (IFN-γ1), and tumor necrosis factor-*α* (TNF-α) in different organs of grass carp were quantified using qPCR. Total RNA was isolated using the TransZol Up Plus RNA Kit (TransGen Biotech, China), with RNA integrity verified by agarose gel electrophoresis (1% w/v) and quantified via spectrophotometric analysis (NanoDrop 2000, OD_260_/OD280:1.8–2.1). cDNA synthesis was performed with 1 μg total RNA using the PrimeScript™ RT Reagent Kit with gDNA Eraser (Takara Bio, Japan), incorporating genomic DNA elimination.

qPCR amplification was conducted on a LightCycler 480 II system (Roche Diagnostics, Germany) using SYBR® Green Premix Pro Taq HS qPCR Kit II (Accurate Biotechnology, China) in 20 μL reactions. Thermal cycling conditions included: 95 °C for 30 s; 40 cycles of 95 °C for 5 s, 57 °C for 30 s, and 78 °C for 5 s. All reactions contained ROX passive reference dye and were performed in triplicate technical replicates. Standard curves for each target gene were established using 10-fold serial dilutions of plasmids containing the corresponding target gene fragments. Transcript abundance was normalized against elongation factor-1α (EF-1α), which was used as the reference gene (Su et al., 2011; Ye et al., 2010). Relative expression levels were determined by comparing the normalized transcript abundance of the experimental groups with that of the control group. Primer pairs (Table 1) were validated for specificity via melt curve analysis.

**Table 1:** Primer sequences of the genes used for qPCR.

Primer	Forward sequence	Reverse sequence	Amplicon(bp)
IL-15	TGCAGGCACTGAAAGACCTC	TTCTGGTTTTGTTGGGTC	179
IL-1β	TCTCCTCGTCTGCTGGGTGT	CAAGACCAGGTGAGGGGAAG	189
IL-6	CAGCAGAATGGGGGAGTTATC	CTCGCAGAGTCTTGACATCCTT	134
IL-8	TCTACCCTCCTAGCCCTCACTG	TCATGGTGCTTTGTTGGCAAGGA	140
IL-10	GCAACAGAACATCAATAGTCCTT	CACCCTTTTCCTTCATCTTTTCA	251
TGF-β1	TTGGGACTTGTGCTCTAT	AGTTCTGCTGGGATGTTT	173
IFN-γ1	CTGAACCAGCTGAATTCCAAG	CTGAGTTCATTTCCTGAAGCTC	130
TNF-α	GCAACTGGGCTCAAGCTTAC	CGGAAATTCGCATAACTGCGT	185
EF-1α	CAGCACAAACATGGGCTGGTTC	ACGGGTACAGTTCCAATACCTCCA	180

### 2.4 16S rRNA amplicon sequencing and bioinformatic analysis

Intestinal DNA was extracted using the E. Z. N. A.® Stool DNA Kit (Omega Bio-tek, USA) according to the manufacturer’s instructions, with nuclease-free water used as a blank control. The extracted DNA was eluted in 50 μL elution buffer and stored at −80 °C prior to analysis. DNA concentration and quality were evaluated before PCR amplification.

The bacterial 16S rRNA V3–V4 regions were amplified using primers 341F (5′-CCTACGGGNGGCWGCAG-3′) and 805R (5′-GACTACHVGGGTATCTAATCC-3′), with sample-specific barcodes attached to the 5′ ends of the primers. PCR amplification was performed in a 25 μL reaction system containing 25 ng template DNA, 12.5 μL PCR Premix, 2.5 μL of each primer, and PCR-grade water. The PCR cycling conditions were as follows: initial denaturation at 98 °C for 30 s; 32 cycles of 98 °C for 10 s, 54 °C for 30 s, and 72 °C for 45 s; followed by a final extension at 72 °C for 10 min. PCR products were verified by 2% agarose gel electrophoresis, purified, quantified, and used for amplicon library construction. Library size and concentration were assessed using Agilent and Illumina platforms, respectively.

Libraries were sequenced on the Illumina HiSeq 6,000 platform (Illumina, San Diego, CA, USA). Raw reads were quality-filtered using fqtrim and chimeric sequences were removed using VSEARCH (v2.14.2). After dereplication and denoising with the DADA2 (v1.16) pipeline, Amplicon Sequence Variants (ASVs) and corresponding feature tables were generated. Taxonomic annotation was performed using the QIIME2 platform (v2020.2) against the SILVA database (132).

Bioinformatics analyses were conducted using QIIME2. Alpha-diversity indices (Sobs, Chao1, ACE, Shannon, and Simpson) and beta-diversity metrics were calculated after rarefaction to the same sequencing depth across samples. Principal Coordinates Analysis (PCoA) based on Bray–Curtis distances and Non-metric Multidimensional Scaling (NMDS) based on weighted and unweighted UniFrac distances were performed to evaluate differences in microbial community structure among groups. Permutational multivariate analysis of variance (PERMANOVA) was used to assess statistical significance among microbial communities. Relative abundances were used for taxonomic analyses, and data visualization was performed using R software (v3.6.1).

Venn diagram analysis was conducted to identify shared and unique ASVs among groups. LEfSe (Linear Discriminant Analysis Effect Size) analysis was performed using the Galaxy LEfSe workflow to identify differentially abundant taxa. The Kruskal–Wallis rank-sum test was first used for multi-group comparisons, followed by pairwise Wilcoxon rank-sum tests for between-group comparisons. Taxa with a logarithmic LDA score > 3.0 and *p* < 0.05 were considered significantly differentially abundant.

Functional prediction of microbial communities was performed using PICRUSt2 (Phylogenetic Investigation of Communities by Reconstruction of Unobserved States 2, v2.3.0-b), and Kyoto Encyclopedia of Genes and Genomes (KEGG) pathway annotations were used to infer potential microbial functions.

### 2.5 Data analysis

All experiments included biological replicates (n ≥ 3). Data normality and homogeneity of variance were assessed using the Shapiro–Wilk test and Levene’s test, respectively. Statistical analyses were performed using one-way ANOVA followed by Tukey’s multiple comparison test in SPSS 20.0. Differences were considered statistically significant at *p* < 0.05. Figures were generated using GraphPad Prism 8.0, and data are presented as mean ± SD.

## 3 Results

### 3.1 Survival rates

**Figure 1:**
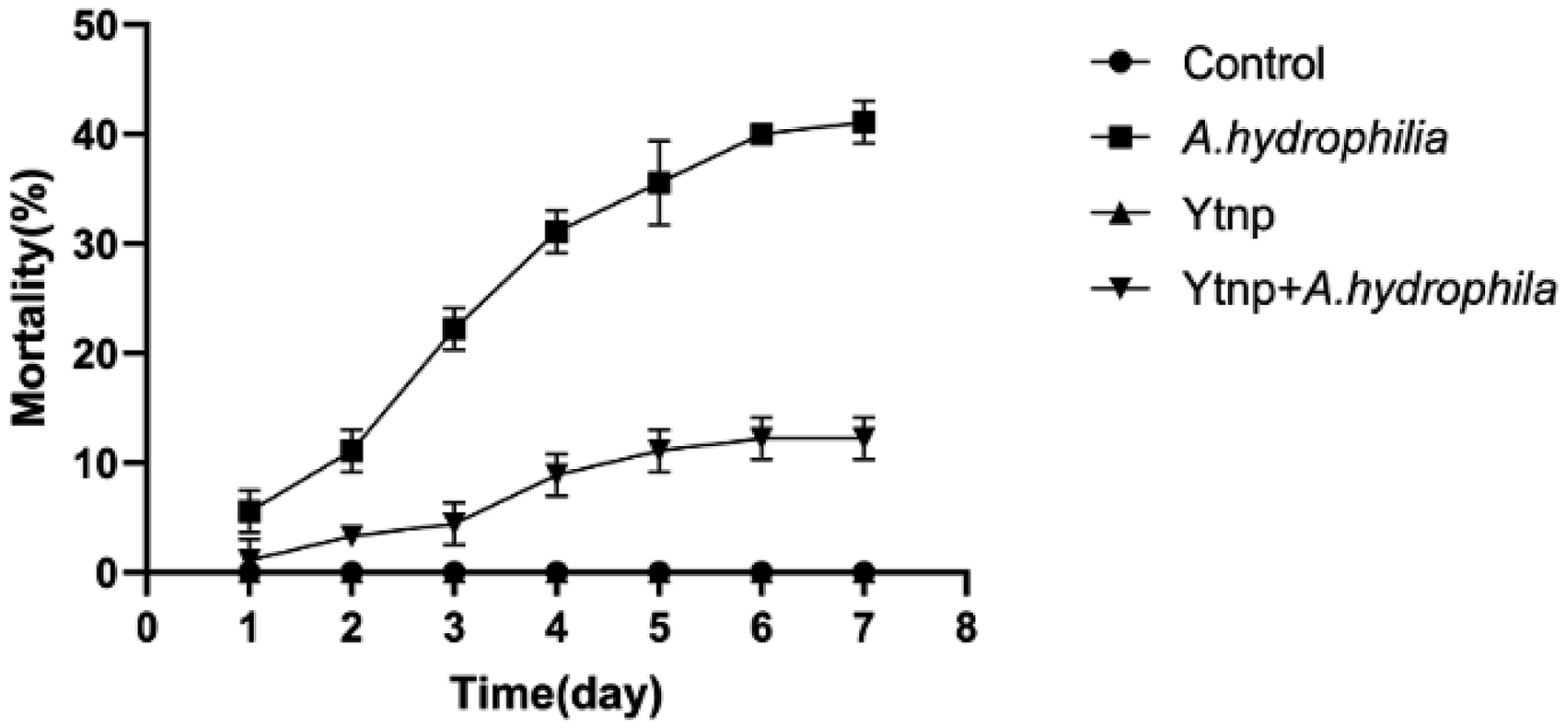
The mortality rates of grass carp in the control group, AH group, YtnP group, and AY group within 7 days.

During the observation period, the survival rate of grass carp was 100% in both the Control and YtnP groups. In contrast, survival rates were 57 and 87% in the AH and AY groups, respectively, with the AY group showing a 30% reduction in mortality compared to the AH group (Figure 1).

### 3.2 Dynamics of immune-related gene expression

**Figure 2:**
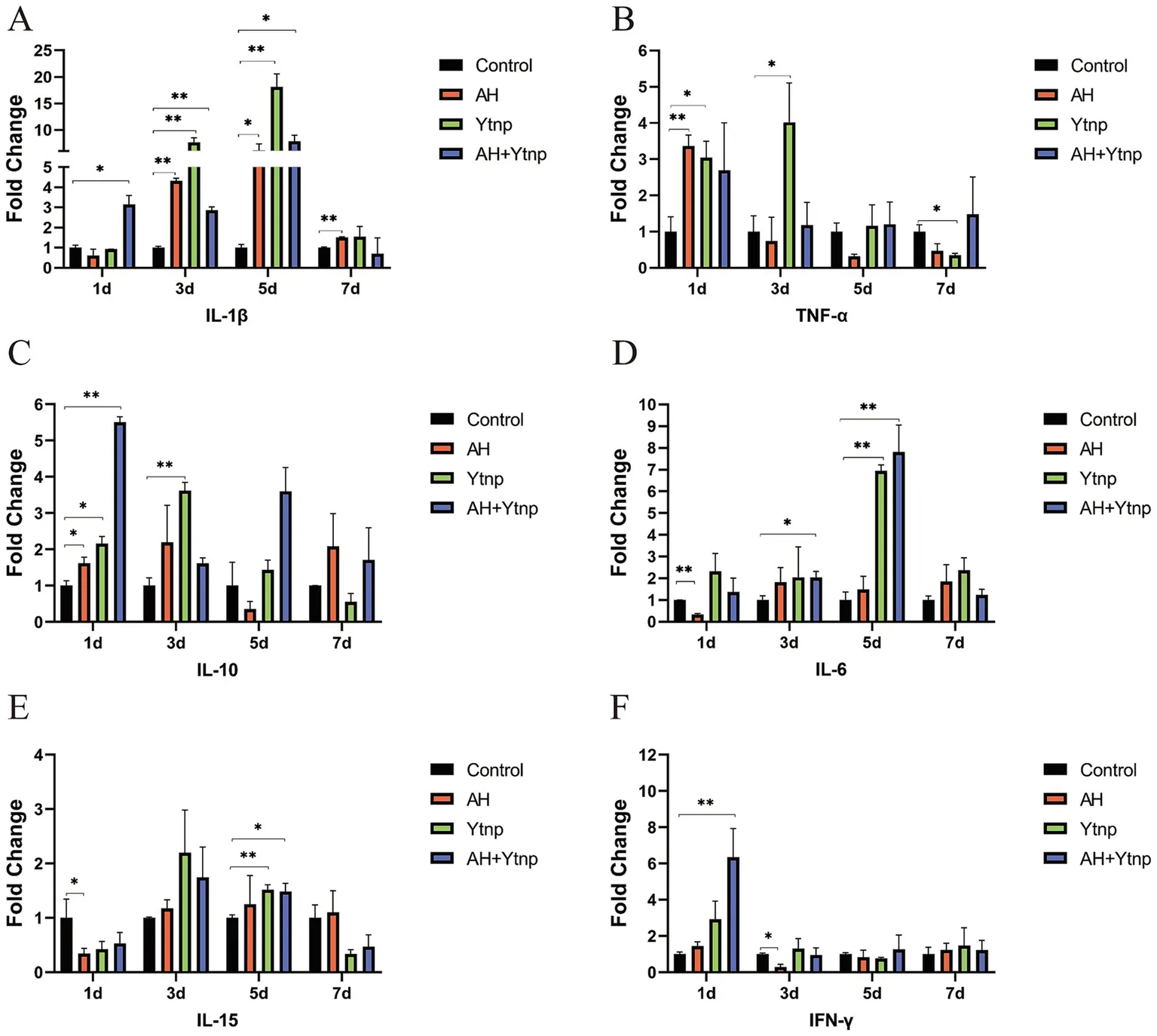
Relative expression levels of inflammatory cytokine genes in the intestines of grass carp from the Control, AH, AY, and YtnP groups: **(A)** IL-1β, **(B)** TNF-*α*, **(C)** IL-10, **(D)** IL-6, **(E)** IL-15, and **(F)** IFN-γ1. *p < 0.05 and **p < 0.01 indicate statistically significant differences.

In the intestine, compared to the control group, IL-1β and TNF-*α* in the AH group exhibited an initial significant increase in relative expression levels, followed by no significant changes. A similar trend was observed for IL-1β, TNF-*α*, IL-10, IL-6, IL-15, and IFN-γ1 in the YtnP group. Compared to the AH group, the AY group showed significantly elevated expression of IL-1β, IL-10, IL-6, and IFN-γ1 initially, with no subsequent differences (Figure 2).

**Figure 3:**
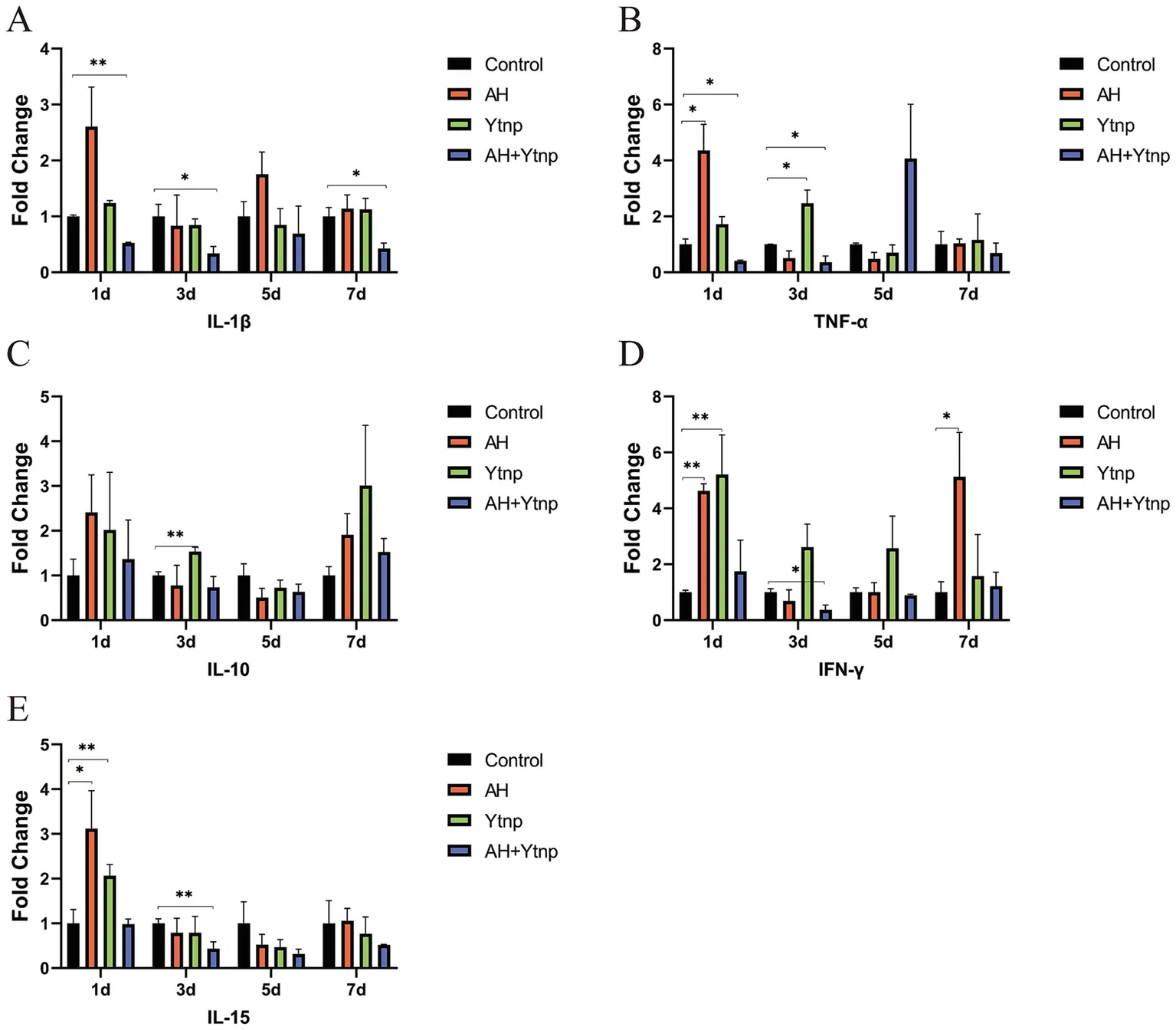
Relative expression levels of inflammatory cytokine genes in the head kidney of grass carp from the Control, AH, AY, and YtnP groups: **(A)** IL-1β, **(B)** TNF-α, **(C)** IL-10, **(D)** IFN-γ1, and **(E)** IL-15. *p < 0.05 and **p < 0.01 indicate statistically significant differences.

In the head kidney, compared to the control group, the AH group displayed an initial significant rise in IL-1β, TNF-*α*, IFN-γ1, and IL-15 expression, followed by stabilization. The YtnP group mirrored this trend for IFN-γ1 and IL-15. Relative to the AH group, the AY group exhibited transient upregulation of IL-1β, TNF-*α*, IFN-γ1, and IL-15, which later normalized to levels comparable to the control group (Figure 3).

**Figure 4:**
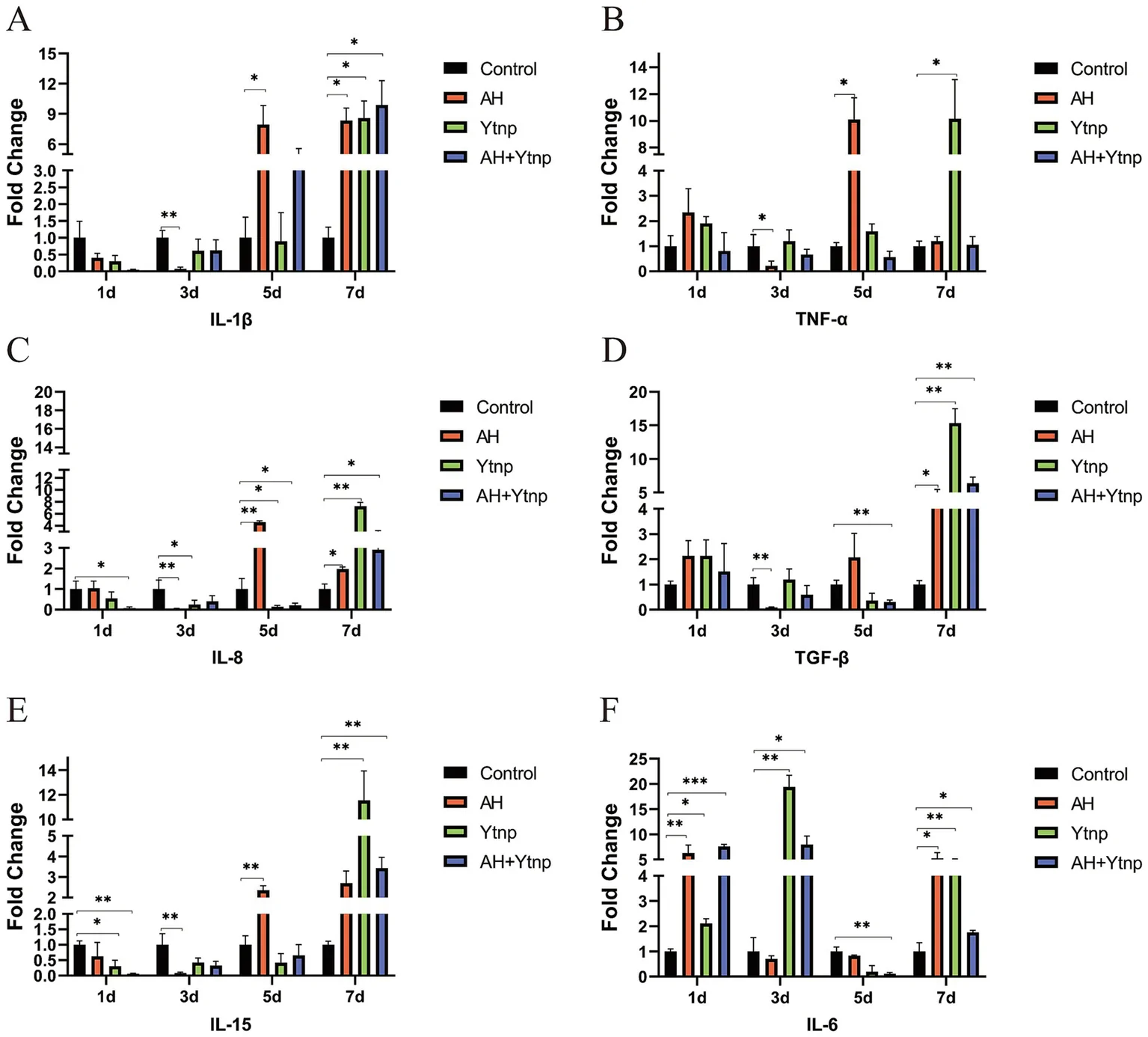
Relative expression levels of inflammatory cytokine genes in the spleen of grass carp from the Control, AH, AY, and YtnP groups: **(A)** IL-1β, **(B)** TNF-α, **(C)** IL-8, **(D)** TGF-β1, **(E)** IL-15, and **(F)** IL-6. *p < 0.05 and **p < 0.01 indicate statistically significant differences.

In the spleen, compared to the control group, IL-1β, TNF-α, IL-8, and TGF-β1 in the AH group showed no initial differences but significantly increased later. At the same time, IL-1β, TNF-α, IL-8, TGF-β1, IL-6, and IL-15 followed a similar delayed increase in the YtnP group. In contrast, the AY group demonstrated no early differences but a subsequent decline in IL-1β, TNF-α, and IL-15 expression compared to the AH group (Figure 4). Specifically, at day 7 post-injection, the expression levels of IL-1β and TNF-α in the spleen of the AY group were significantly lower than those in the AH group (*p* < 0.05).

Collectively, these results indicate that YtnP treatment altered the expression patterns of multiple immune-related genes during *A. hydrophila* infection.

### 3.3 Intestinal microbial diversity and richness

**Figure 5:**
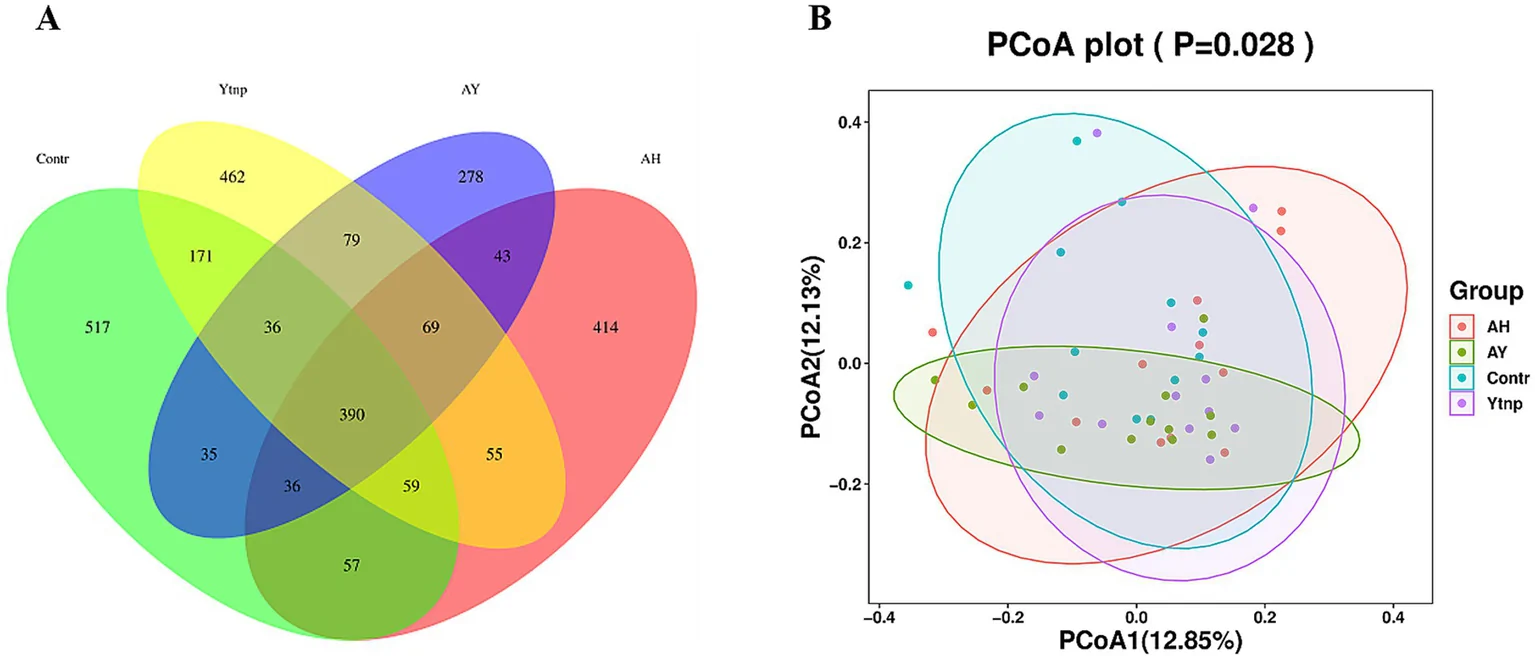
Comparison of the intestinal microbial communities among the Control, AH, AY, and YtnP groups **(A)** Venn diagram showing the shared and unique amplicon sequence variants (ASVs). **(B)** Principal coordinates analysis (PCoA) based on beta-diversity indices.

After quality control and read assembly, 3,521,050 effective tags were retained from 3,892,561 raw reads, accounting for more than 90% of the raw reads. All ASVs with an average abundance greater than 1 were selected for Venn diagram analysis to identify core and unique ASVs among groups. A total of 390 ASVs were shared among all intestinal samples according to the Venn diagram analysis. In contrast, 517, 462, 278, and 414 unique ASVs were identified in the Control, AH, YtnP, and AY groups, respectively (Figure 5A). The YtnP group exhibited the lowest number of unique ASVs, whereas the Control group showed the highest number of unique ASVs.

Assessment of alpha-diversity indices (Chao1, Shannon, and Simpson) revealed no significant differences in intestinal microbial richness or diversity among groups (*p* > 0.05) (Supplementary Figure S1). Non-metric multidimensional scaling (NMDS) based on Unweighted and Weighted UniFrac distances was performed to visualize beta diversity. Samples from the Control, AH, YtnP, and AY groups clustered closely in the NMDS plots, suggesting limited separation in microbial community composition across groups. PERMANOVA analysis based on Unweighted UniFrac distances confirmed no significant differences among the four groups (*p* > 0.05), and similar results were obtained using Weighted UniFrac distances (*p* > 0.05) (Supplementary Figure S2). Principal Coordinates Analysis (PCoA) based on weighted Bray–Curtis distances showed that PCoA1 and PCoA2 explained 12.85 and 12.13% of the total variation, respectively (Figure 5B).

### 3.4 The microbial community composition in the intestine

**Figure 6:**
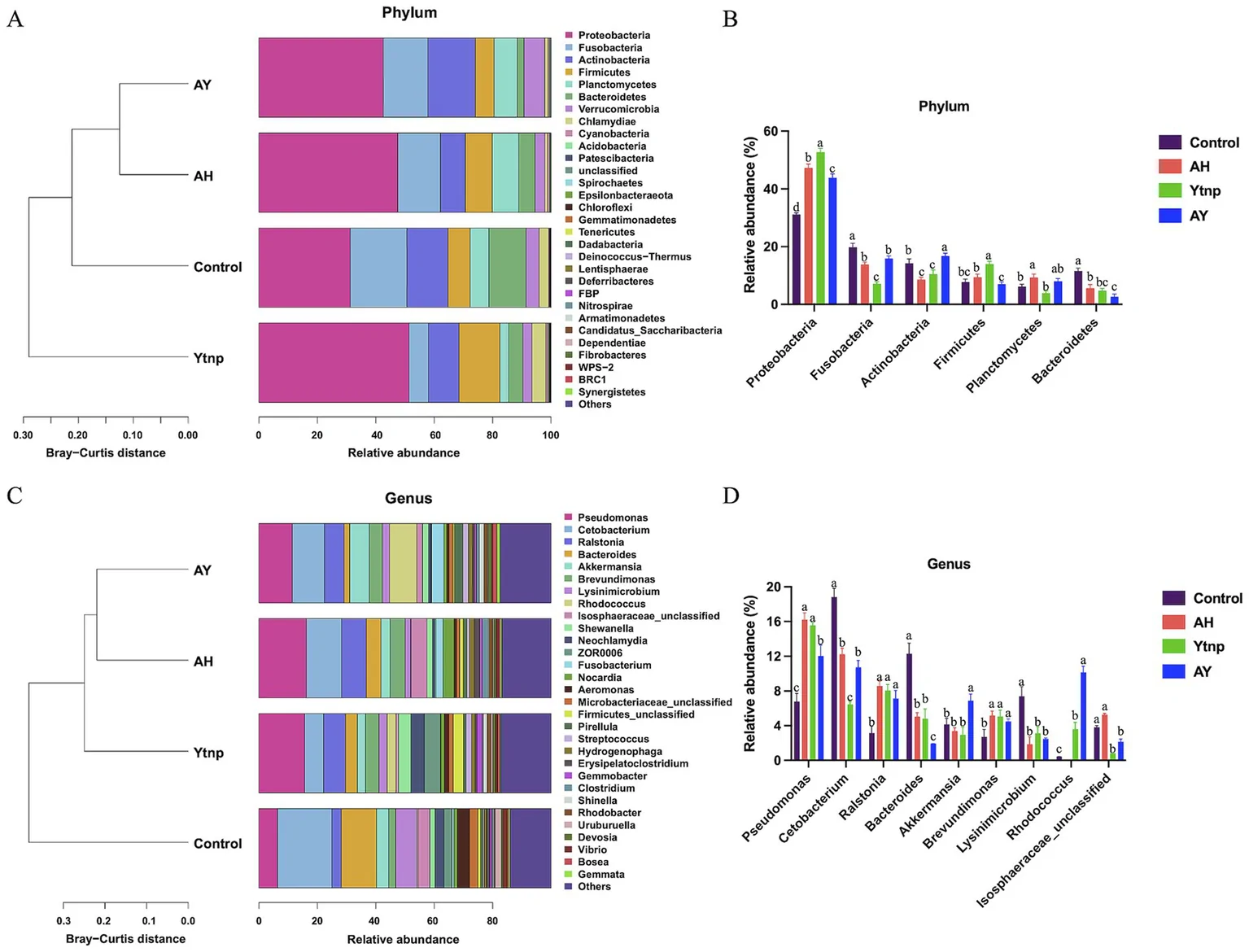
Relative abundance and differential analysis of the intestinal microbiota at the phylum and genus levels in grass carp from the Control, AH, AY, and YtnP groups. **(A)** Relative abundance at the phylum level. **(B)** Differential analysis at the phylum level. **(C)** Relative abundance at the genus level. **(D)** Differential analysis at the genus level. Different letters represent significant differences observed between groups (*p* < 0.05).

At the phylum level, the intestinal microbiota community of grass carp was dominated by *Proteobacteria* (31.24%), *Fusobacteria* (19.45%), *Actinobacteria* (14.01%), and *Bacteroidetes* (12.70%). Based on the relative abundance, the dominant phyla of the grass carp gut microbiota in the AH group were *Proteobacteria* (47.52%), *Fusobacteria* (14.63%), *Firmicutes* (9.21%), and *Planctomycetes* (9.07%). In contrast, the YtnP group was characterized by *Proteobacteria* (51.33%), *Firmicutes* (13.99%), *Actinobacteria* (10.44%), and *Fusobacteria* (6.7%), whereas *Proteobacteria* (42.57%), *Actinobacteria* (16.25%), *Fusobacteria* (15.27%), and *Planctomycetes* (7.95%) dominated the AY group (Figure 6A). The AH group demonstrated significantly increased *Proteobacteria* and decreased *Fusobacteria* relative abundances compared with the Control group (*p* < 0.05), whereas the AY group exhibited a reversal of these phylum-level alterations. *Actinobacteria* abundance was significantly reduced in the AH group but restored in the AY group versus the Control group (*p* < 0.05). Compared to the AH groups, the AY group showed significant reductions in *Firmicutes* and *Bacteroidetes* abundance (*p* < 0.05) (Figure 6B).

The top 10 bacterial genera common to all groups comprised *Pseudomonas*, *Cetobacterium*, *Ralstonia*, *Bacteroides*, *Akkermansia*, *Brevundimonas*, *Lysinimicrobium*, *Rhodococcus*, *unclassified_ Isosphaeraceae*, and *Shewanella* (Figure 6C). All experimental groups exhibited significantly elevated *Pseudomonas*, *Ralstonia*, *Brevundimonas*, and *Rhodococcus* abundance relative to the Control group, concurrent with reduced *Cetobacterium*, *Bacteroides*, and *Lysinimicrobium* levels (*p* < 0.05). In contrast, the relative abundance of *Pseudomonas*, *Bacteroides*, and *unclassified_Isosphaeraceae* was significantly decreased in YtnP and AY groups compared to the AH group (*p* < 0.05) (Figure 6D).

### 3.5 Significantly different species identified by LEfSe

**Figure 7:**
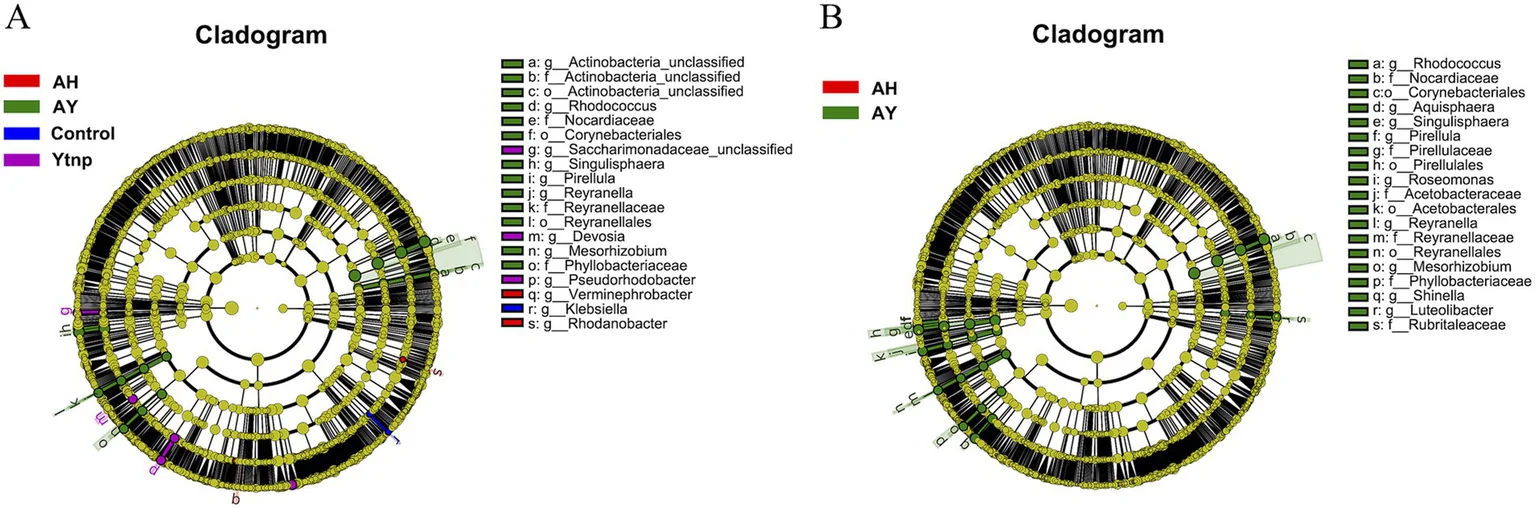
Identification of differentially abundant intestinal bacterial taxa by LEfSe analysis. **(A)** Multi-group comparison among the Control, AH, AY, and YtnP groups. **(B)** Pairwise comparison between the AH and AY groups. Only taxa with an LDA score > 3 and *p* < 0.05 are shown.

LEfSe analysis was performed to identify differentially abundant bacterial taxa among the experimental groups. Multi-group comparisons identified *Verminephrobacter* and *Rhodanobacter* as AH group-specific biomarkers with elevated abundance, while *Actinobacteria*_*unclassified*, *Rhodococcus*, *Singulisphaera*, *Pirellula*, *Reyranella*, and *Mesorhizobium* were enriched in the AY group (Figure 7A). Pairwise analysis between AY and AH groups revealed significantly higher abundance of *Rhodococcus*, *Aquisphaera*, *Singulisphaera*, *Pirellula*, *Roseomonas*, *Reyranella*, *Mesorhizobium*, *Shinella*, and *Luteolibacter* in AY, whereas no taxa demonstrated significant abundance predominance in AH (Figure 7B).

### 3.6 Predicted intestinal microbial function

**Figure 8:**
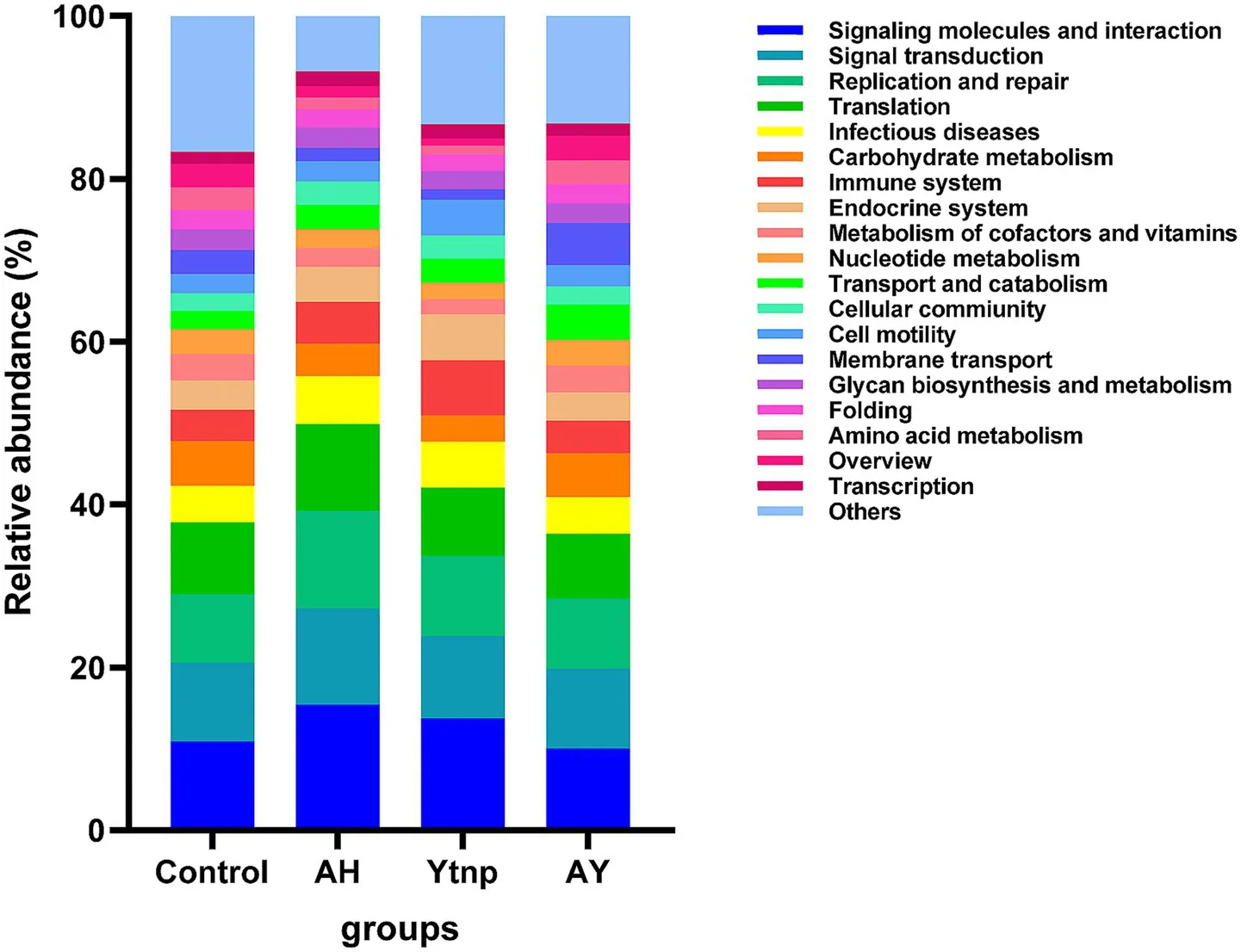
KEGG annotation analysis of intestinal microbial functions.

**Figure 9:**
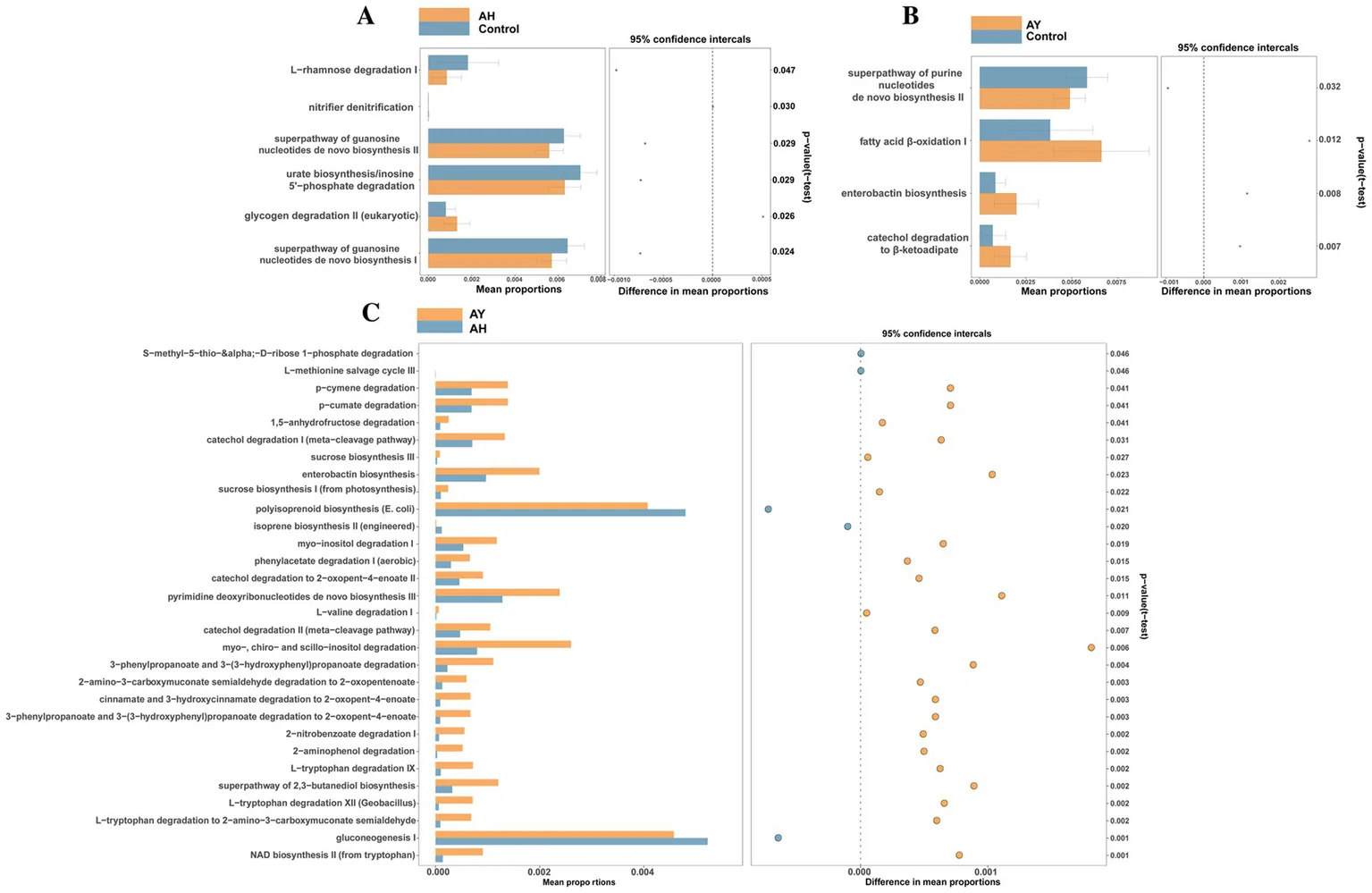
Predicted functional pathways of the intestinal microbiota showing significant differences among experimental groups. **(A)** Comparison between the AH and Control groups. **(B)** Comparison between the AY and Control groups. **(C)** Comparison between the AH and AY groups.

The KEGG function of ASVs in each group was predicted using PICRUSt2, revealing predicted predominant functional categories: Signaling molecules and interaction, Signal transduction, Replication and repair, Translation, Infectious diseases, and Carbohydrate metabolism (Figure 8). Compared to the control group, the AH group was predicted to show upregulation of Carbohydrate metabolism, Nucleotide metabolism, and Antioxidant metabolism, suggesting potential changes in microbial functional profiles related to cellular proliferation, immune activation, and energy demand (Figure 9A). The AY group exhibited elevated predicted levels of Lipid metabolism, Enterobactin biosynthesis, and Aromatic compound metabolism compared to the control group (Figure 9B). Comparative analysis between the AH and AY groups revealed significant predicted upregulation of Aromatic compound, Carbohydrate, and Nucleotide metabolism in the AY group, with concurrent downregulation of Polyisoprenoid biosynthesis and Gluconeogenesis I (Figure 9C).

## 4 Discussion

This study established an *A. hydrophila* infection model in grass carp. Exogenous administration of YtnP alone did not affect fish survival rates, but significantly improved survival upon *A. hydrophila* challenge. YtnP may suppress bacterial virulence through quorum-quenching activity, thereby contributing to enhanced host resistance. Oral administration of *Bacillus*-derived AHL lactonase in feed significantly mitigated *A. hydrophila* infections in zebrafish, representing the first report of oral AHL lactonase efficacy against this pathogen (Cao et al., 2012). YtnP treatment also substantially improved survival in *P. aeruginosa* PAO1-infected zebrafish (Djokic et al., 2022) and extended lifespan in *P. aeruginosa* MMA83-infected *Caenorhabditis elegans* (Curcic et al., 2024). These findings collectively demonstrate that quorum quenching enzymes constitute an effective strategy against aeromonad diseases in fish.

Endogenous signaling molecules tightly control inflammatory responses to prevent self-damage and excessive inflammation (Ruby et al., 2011). Pathogens respond to host immune threats by interfering with inflammatory signaling molecules to establish persistent infections (Reddick and Alto, 2014). To evaluate the immunomodulatory effects of YtnP during *A. hydrophila* infection, we focused on a panel of representative innate immune markers in grass carp. IL-1β, TNF-*α*, IL-8, and IL-6 are key pro-inflammatory cytokines involved in pathogen recognition, leukocyte recruitment, and inflammatory signal amplification. IL-15 participates in immune cell activation and proliferation, whereas TGF-β1 functions as an important immunoregulatory cytokine that contributes to inflammation resolution and tissue repair. Immune gene profiling showed early upregulation of pro-inflammatory IL-1β and TNF-α in the AH group (*p* < 0.05), indicating an acute inflammatory response to pathogen invasion, which is similar to Song’s study, where grass carp gut inflammation was induced by 2.7 × 10^6^ cfu/mL of *A. hydrophila* (Song et al., 2014). Overall, compared to the AH group, the AY group exhibited significant upregulation of IL-6, and IL-15 in the intestine and head kidney, along with downregulation of IL-1β and TNF-α in the spleen at later stages (day 7), showing YtnP-mediated multifactorial changes during AH infection (*p* < 0.05). IFN-*γ* immune adjuvants can boost immune responses and confer strong protection against chronic enteritis (Wang et al., 2021). IL-6 and IL-15 are crucial for immune regulation during pathogen invasion (Costa et al., 2011; Wu et al., 2022). In the AY group, the concurrent induction of selected pro-inflammatory cytokines and anti-inflammatory mediators suggests that YtnP may contribute to maintaining a balance between pathogen clearance and inflammatory control.

Notably, the expression profiles of immune-related genes varied among the intestine, spleen, and head kidney, reflecting the tissue-specific nature of the host response to *A. hydrophila* infection. As the primary site of pathogen exposure, the intestine rapidly activates local inflammatory responses to limit bacterial colonization, whereas the spleen mainly coordinates systemic immune surveillance and inflammatory regulation (Salinas, 2015). The head kidney, a major hematopoietic and immune organ in teleosts, contributes to immune cell activation and recruitment during infection (Bjørgen and Koppang, 2021; Geven and Klaren, 2016). The stronger pro-inflammatory responses in the intestine may be associated with direct bacterial contact and activation of local innate immune signaling pathways, whereas the more moderate responses in the spleen and head kidney may reflect their roles in systemic immune integration rather than frontline pathogen sensing (Magnadóttir, 2006; Whyte, 2007). The differential expression of pro-inflammatory cytokines (IL-1*β*, TNF-*α*, IL-8, and IL-6), together with the immunoregulatory cytokines IL-15 and TGF-β1, suggests that host defense involves coordinated interactions between local mucosal immunity and systemic immune regulation. Moreover, the ability of YtnP to attenuate excessive inflammatory responses while promoting immune homeostasis may be associated with both quorum-sensing disruption and microbiota-mediated immunomodulation (Guo et al., 2023).

Upon hatching, fish begin to acquire intestinal microbial communities, establishing a host-beneficial microecological environment (Liu et al., 2021). In the present study, although α-diversity indices showed no significant differences among groups, β-diversity analysis revealed a tendency toward separation in the PCoA plot, suggesting structural reorganization rather than simple abundance shifts in the grass carp intestinal microbiota. However, this trend was not statistically supported by PERMANOVA, and NMDS analysis showed close clustering of samples across groups. Collectively, these results indicate that the treatments did not significantly alter the overall community structure, which remained largely stable. Dominant phyla in the intestinal microbiome included *Proteobacteria*, *Fusobacteria*, *Actinobacteria*, and *Bacteroidetes*, differing from previous reports, potentially due to environmental variations (Wu et al., 2013). Compared to controls, *Proteobacteria* abundance significantly increased in the AH group (*p* < 0.05), accompanied by Pseudomonas expansion and altered expression of splenic IL-8 and TGF-β1, although the relationships among these changes remain unclear. In contrast, the AY group exhibited restored *Fusobacteria* abundance, which may contribute to immune homeostasis through the production of metabolites such as short-chain fatty acids (Yang et al., 2020). *Actinobacteria* abundance also rebounded in the AY group, with significant enrichment of *Rhodococcus*, a genus that has been associated with antimicrobial activity and potential immunomodulatory functions (Ghanei-Motlagh et al., 2021a,b).

Functional prediction indicated enriched pathways for Infectious diseases and Signal transduction in the AH group, aligning with pathogen-driven inflammation, whereas the AY group displayed enhanced Amino acid metabolism and Membrane transport, suggesting microbiota support for metabolic demands to promote anti-inflammatory environments. Additionally, the AY group shows significant enrichment in Aromatic compound metabolism, Enterobactin biosynthesis, and Catechol degradation pathways, which may correlate with beneficial metabolic activities. Enterobactin, produced by symbiotic bacteria, is utilized by the host to maintain its iron homeostasis (Qi and Han, 2018). *Salmonella* and enterohemorrhagic *Escherichia coli* use the catecholamine sensor QseC to detect AI-3 (Moreira and Sperandio, 2012), regulating flagellar motility and virulence gene expression. Catecholamines are catechol derivatives, and AI-3 is a quorum sensing signal molecule (Sperandio et al., 2003). Notably, emerging evidence suggests that quorum sensing systems are highly interconnected, with multiple signal–response pathways forming complex regulatory networks in Gram-negative bacteria (Papenfort and Bassler, 2016). For example, AI-3, a host–microbe interkingdom signaling molecule, is sensed by the QseC sensor kinase in pathogens such as *Salmonella* and enterohemorrhagic *Escherichia coli*, regulating virulence gene expression and motility (Sperandio et al., 2003; Moreira and Sperandio, 2012). Although YtnP specifically degrades AHL signals, the observed enrichment of catechol-related metabolic pathways in the AY group suggests a potential indirect influence on AI-3-associated signaling networks, possibly through microbiota-mediated metabolic modulation. However, it should be noted that YtnP does not directly target AI-3 or other non-AHL signaling molecules, and its effects on these systems are likely indirect and context-dependent. Previous studies have demonstrated that quorum quenching enzymes primarily exhibit specificity toward AHL-type signals (Grandclément et al., 2015), indicating that cross-system regulatory effects remain hypothetical and require further experimental validation. Therefore, future studies integrating multi-omics approaches and signal molecule quantification are necessary to elucidate the broader ecological impact of YtnP on complex quorum sensing networks.

Building upon these observations, YtnP appears to exert anti-infective effects through both direct and indirect mechanisms. First, YtnP directly attenuates AHL-mediated quorum sensing by degrading *A. hydrophila*-derived signal molecules, suppressing virulence gene expression. This interpretation is consistent with the reduced abundance of *Pseudomonas* and the decreased representation of the pathogenicity-associated taxon *Verminephrobacter* in the AY group. Similarly, the QS inhibitor hordenine disrupts *P. aeruginosa* PAO1 quorum-sensing system, interfering with energy metabolism, amino acid metabolism, and nucleotide metabolism, ultimately attenuating its pathogenicity (Zhou et al., 2019). Second, YtnP enhances host immune tolerance by remodeling microbial communities to favor probiotic colonization, whose metabolites likely activate immune pathways and induce anti-inflammatory factor secretion. Analogously, dietary supplementation with quorum-sensing probiotics protects Asian seabass against *Vibrio harveyi* infection while increasing intestinal beneficial bacteria abundance (Ghanei-Motlagh et al., 2021a,b).

This study provides evidence that YtnP alleviates *A. hydrophila*-induced inflammation and microbiota dysbiosis in grass carp, potentially through a cascade involving quorum-quenching activity, microbiota remodeling, and immune modulation. Compared to conventional antibiotics, its advantage lies in targeting pathogen communication systems without disturbing commensal microbiota, thereby avoiding risks of microbial collapse and secondary infections. These findings support the potential application of anti-virulence strategies based on microbiota–immune interactions as environmentally friendly alternatives for disease management in aquaculture.

## Data Availability

The sequencing data have been deposited in the Sequence Read Archive (SRA) at the National Center for Biotechnology Information (NCBI) under BioProject accession number PRJNA1504855. The original contributions presented in the study are included in the article/Supplementary material, further inquiries can be directed to the corresponding author.
