# Supplementary material for: Effect of quorum quenching enzyme YtnP on immune response and intestinal microbiota in Aeromonas hydrophila-infected grass carp (Ctenopharyngodon idella)
Source: Front Microbiol. 2026 Aug 24;17:1853827. doi: 10.3389/fmicb.2026.1853827 (PMC13547058; doi:10.3389/fmicb.2026.1853827)
Supplement: Supplementary file 1 [file Supplementary_file_1.DOCX]

Supporting Information

Figure S1

Figure S1. Alpha diversity analysis of intestinal microbiota based on 16S rRNA gene amplicon sequencing.

Alpha diversity was compared among four groups (Control, AH, YtnP, and AY) using four indices: Observed OTUs (A), Chao1 (B), Shannon (C), and Simpson (D). Data are presented as violin plots, with P-values from Kruskal-Wallis tests shown. No significant differences were observed among the four groups for all indices (*P* > 0.05).

Figure S2

Figure S2. **Beta diversity analysis of** intestinal **microbiota based on 16S rRNA gene amplicon sequencing: NMDS ordination plots**
Non-metric multidimensional scaling (NMDS) analysis was performed on four groups (Control, AH, YtnP, and AY) based on Unweighted UniFrac (A) and Weighted UniFrac (B) distance matrices. Each point represents an individual sample, and the distance between points reflects the similarity of microbial community structures. Stress values are 0.15 (A) and 0.13 (B), indicating good representativeness of the ordination results. PERMANOVA (999 permutations) revealed no significant differences in either analysis (Unweighted: F = 58.77, *P* = 0.2112, R² = 0.800; Weighted: F = 54.28, *P* = 0.2943, R² = 0.787).
